# INVICTUS II: comparison of disc diffusion and broth microdilution methods for susceptibility testing of ceftolozane/tazobactam and five comparator agents against a subset of INVICTUS I *Pseudomonas aeruginosa* clinical isolates

**DOI:** 10.1093/jacamr/dlag212

**Published:** 2026-10-08

**Authors:** Kate Baldwin, Adela Alvarez-Buylla, Michael Allen, Ricky Henry-Grant, Edita Custic Weguelin, Mandy Wootton

**Affiliations:** Specialist Antimicrobial Chemotherapy Unit, Public Health Wales NHS, Cardiff, UK; Medical Affairs, MSD (UK) Limited, 120 Moorgate, London EC2M 6UR, UK; Medical Affairs, MSD (UK) Limited, 120 Moorgate, London EC2M 6UR, UK; Medical Affairs, MSD (UK) Limited, 120 Moorgate, London EC2M 6UR, UK; Medical Affairs, MSD (UK) Limited, 120 Moorgate, London EC2M 6UR, UK; Specialist Antimicrobial Chemotherapy Unit, Public Health Wales NHS, Cardiff, UK

## Abstract

**Objectives:**

A subset of clinical *Pseudomonas aeruginosa* isolates from the INVICTUS I study was selected to compare antimicrobial susceptibility performance for disc diffusion and broth microdilution of ceftolozane/tazobactam and five comparator antipseudomonal agents.

**Methods:**

Overall, 159 non–cystic fibrosis isolates were chosen from INVICTUS I that had the smallest zone diameters from disc testing to ceftolozane/tazobactam, including isolates that were close to the breakpoints. The isolates were tested across 14 centres in the UK. Susceptibility results were interpreted using EUCAST 2026 criteria.

**Results:**

Discrepancy analysis showed combined error rates (very major and major errors) highest for ceftolozane/tazobactam, ceftazidime and piperacillin/tazobactam (15.7%, 14.5% and 12.6%, respectively); when adjusted to remove isolates close to the breakpoint for ceftolozane/tazobactam and piperacillin/tazobactam there were still remarkably high error rates (10.6% and 7.2%, respectively). Essential agreement and bias analysis were not performed as MICs were being compared with zone diameters.

**Discussion:**

Our findings highlight substantial challenges in performing and interpreting susceptibility testing for these two important antipseudomonal β-lactam/β-lactamase inhibitors, which are commonly employed as either first-line empirical therapy (piperacillin/tazobactam) or for serious and MDR infection (ceftolozane/tazobactam). A review of disc testing for these agents may be warranted, and diagnostic laboratories may wish to review the technical limitations and put in place mitigating measures.

## Introduction

*Pseudomonas aeruginosa* remains an important pathogen of global healthcare concern, particularly in the intensive care setting where ventilator-associated pneumonia and bloodstream infection associated with *P. aeruginosa* are linked to increased morbidity and mortality.^1^ Patients with cystic fibrosis (CF) are particularly susceptible to this opportunistic pathogen, resulting in chronic airway infections in up to 60%–70% of adults with CF.^1^ Treatment of *P. aeruginosa* infections remains a persistent clinical challenge.^1^ Significant intrinsic resistance coupled with increasing levels of acquired resistance has resulted in an increase in MDR phenotypes, driving the need for novel antipseudomonals.^2,3^

Data from the ECDC EARS-Net 2024 report show that of the 20 565 invasive *P. aeruginosa* isolates, 10% demonstrated combined resistance to ≥3 antimicrobial groups (among piperacillin/tazobactam, ceftazidime, carbapenems, fluoroquinolones and aminoglycosides).^2^ This represents a downward trend from 2020 to 2024 but nevertheless presents a concerning landscape.

Fluoroquinolones, aminoglycosides and polymyxins have traditionally formed the mainstay of antipseudomonal agents, the latter two of which are accompanied with an increased risk of nephrotoxicity. Ceftolozane/tazobactam, a relatively novel cephalosporin/β-lactamase inhibitor, is generally well tolerated even by those patients with renal impairment, and demonstrates potent *in vitro* activity against MDR *P. aeruginosa*, where it has been shown to be the most active β-lactam antibiotic available.^4,5^ High susceptibility rates in comparison with other β-lactam/β-lactamase-inhibitor combinations, fluoroquinolones and even carbapenems, coupled with potent activity against isolates demonstrating up-regulated efflux and with derepressed *AmpC*, make ceftolozane/tazobactam an attractive option for treatment of MDR *P. aeruginosa*.^4^

The EUCAST disc diffusion (DD) method is a well-established and easy-to-perform method used in many European diagnostic laboratories as a first-line test. It is often the sole method used in these laboratories, owing to its ability to test a large variety of antimicrobial agents and its relative low cost. Susceptibility testing of *P. aeruginosa* can be challenging due to its various morphotypes, especially problematic in isolates from CF patients.^3^

Results from isolates tested in the previous INVICTUS I study showed 90.8% (*n* = 1209) susceptibility to ceftolozane/tazobactam (after retesting in the central testing laboratory), with the lowest coefficient of variation for disc diameter measurement reproducibility.^3^ The purpose of this present study was to evaluate the accuracy of locally performed DD results using latest EUCAST interpretative criteria for ceftolozane/tazobactam and five other antipseudomonal agents by comparing against the MICs from the ‘gold-standard’ broth microdilution (BMD) method on a subset of the INVICTUS I isolates.

## Materials and methods

### Ethics

Only bacterial isolates were included and analysed in the study. No human tissue was collected, stored or analysed and no patient data were recorded, thus ethical approval was not applicable for this study. Health Research Authority (HRA) approval was obtained.

### Methodology

A subset of 159 clinical *P. aeruginosa* isolates from non-CF patients was selected from 1326 isolates collected at the 14 UK laboratories during the original INVICTUS I study (2015–2018). The subset included a wide range of specimen types and represented all ceftolozane/tazobactam phenotypes: i.e. isolates with small zone diameters (i.e. resistant) and large zone diameters (susceptible) plus isolates with zone diameters near to the ceftolozane/tazobactam breakpoint. This selection was designed to robustly evaluate EUCAST DD methodology against the BMD reference method.

Participating laboratories around the UK performed antimicrobial susceptibility testing (AST) using their routine in-house materials including Mueller–Hinton agar and antimicrobial discs from their usual suppliers. This approach was intentionally chosen to reflect the real-life modality and conditions under which AST is performed in UK clinical bacteriology diagnostic laboratories and therefore to assess performance under routine conditions.

Reference BMD testing was performed at a central testing laboratory at the Specialist Antimicrobial Chemotherapy Unit, Public Health Wales, Cardiff, UK.

A comparative analysis to determine categorical agreement between DD and BMD for obtaining MICs was conducted for ceftolozane/tazobactam and five other comparator antibiotics: piperacillin/tazobactam, ceftazidime, imipenem, meropenem and aztreonam. Zone diameter interpretation was updated in accordance with the latest version of EUCAST (version 16, 2026).^6^ Any isolate with a categorical discrepancy between MIC and zone diameter, where the MIC was not within 1 log_2_ dilution either side of the breakpoint, was retested using DD in the central testing laboratory (discs and Mueller–Hinton agar: Oxoid-Thermo Fisher, Hampshire, UK).

BMD was performed to obtain MICs according to ISO 20776-1.^7^ Mueller–Hinton broth (Becton Dickinson, Berkshire, UK) was added to sterile U-shaped 96-well microtitre plates with lids (Greiner Bio-one, Gloucestershire, UK). Ceftolozane/tazobactam, piperacillin/tazobactam, ceftazidime, imipenem, meropenem and aztreonam concentrations ranging from 0.008 to 128 mg/L were used. Inocula were prepared using sterile saline (Oxoid-Thermo Fisher, Hampshire, UK) and Mueller–Hinton broth (Becton Dickinson, Berkshire, UK) to equal 10^5^ cfu/mL within each well. *P. aeruginosa* (ATCC 27853) and *Escherichia coli* (ATCC 35218) were used as the quality control strains to monitor the performance of the tests. Plates were incubated at 35 ± 1°C in air for 16-20 h. The MIC was taken as the first concentration where the highest visible growth reduction occurred. MIC results were interpreted using EUCAST 2026 criteria.^6^

### Statistical analysis

Categorical agreement of the disc method with the reference gold standard method was performed. Sensitivity and specificity were obtained by comparing the percentage of isolates reported as susceptible (S) or resistant (R). Agents for which there was susceptible increased exposure (SIE) or an area of technical uncertainty (ATU) category were included in the susceptible and resistant category, respectively. It is acknowledged that this method of sensitivity and specificity analysis is not as per the ISO 20776-2 standard;^8^ nevertheless, the insight is valuable for comparing performance across centres. The ISO 20776-2 standard^8^ defines a specificity of ≥95% for a qualitative device versus MIC.

Essential agreement and bias analysis were not performed as MICs were being compared with zone diameters. MIC_50_, MIC_90_ and range analysis was performed for each agent to determine any testing bias with the isolates selected for the study. Discrepancies were analysed to deduce very major error (VME) and major error (ME) rates to further analyse performance.

## Results

Of the 159 isolates selected and tested, 136 (86%) were susceptible by BMD to ceftolozane/tazobactam and 23 (15%) were resistant, compared with 117 (74%) susceptible and 42 (26%) resistant by DD. Susceptibility and resistance results for all agents tested are presented in Table 1. It should be noted that these results are based on a selected and therefore biased subset of isolates.

**Table 1.** Summary table of categorical agreement, sensitivity and specificity

	C/T		TZP		CAZ		IPM		MEM		ATM	
	BMD	DD	BMD	DD	BMD	DD	BMD	DD	BMD	DD	BMD	DD
S, *n*	136	117							117	113		
R, *n*	23	42	62	39	54	71	46	48	28	36	58	62
I, *n*			97	114	105	88	113	111	14	10	101	97
ATU, *n*				6								
S, %	86	74							73	71		
R, %	15	26	39	24	34	45	29	30	18	23	37	39
I, %			61	72	66	55	71	70	9	6	64	61
ATU, %				4								
Sensitivity, %	87		63		94		85		100		90	
Specificity, %	84		98		81		92		98		90	

ATM, aztreonam; ATU, area of technical uncertainty; BMD, broth microdilution; CAZ, ceftazidime; C/T, ceftolozane/tazobactam; DD, disc diffusion; I, susceptible increased exposure; IPM, imipenem; MEM, meropenem; R, resistant; S, susceptible; TZP, piperacillin/tazobactam.

Sensitivity and specificity analysis was performed to compare the susceptibility and resistance between DD and the BMD reference method. It is acknowledged that sensitivity and specificity in its strictest sense and according to ISO 2020776-2 uses growth and no growth for analysis.^8^ Nevertheless, sensitivity was highest for meropenem (90%) and ceftazidime (94%), and specificity highest for piperacillin/tazobactam and meropenem (both 98%) (Table 1).

MIC_50_ and MIC_90_ analyses were performed to characterize the MIC distributions of each agent within the selected isolate set (Table 2). Ceftolozane/tazobactam had an MIC_90_ of 8 mg/L, indicating that 90% of isolates were inhibited at relatively low concentrations. In contrast, piperacillin/tazobactam, ceftazidime and aztreonam exhibited MIC_90_ values of 128 mg/L. The broad MIC ranges observed for all agents indicate substantial variability in susceptibility across the isolate set.

**Table 2.** MIC_50_ and MIC_90_ analysis of each agent

	C/T	TZP	CAZ	IPM	MEM	ATM
MIC_50_	1	8	4	2	1	8
MIC_90_	8	128	128	32	32	128
Range	0.5–128	1–128	1–128	0.25–128	0.03–28	0.5–128

ATM, aztreonam; CAZ, ceftazidime; C/T, ceftolozane/tazobactam; IPM, imipenem; MEM, meropenem; TZP, piperacillin/tazobactam.

Figures 1 and 2 show the distribution of DD zone diameters versus BMD MIC for all 159 isolates to ceftolozane/tazobactam and piperacillin/tazobactam. For ceftolozane/tazobactam, isolates formed distinct susceptible and resistant clusters with minimal overlap near the breakpoint, demonstrating good concordance between DD and MIC testing. In contrast, piperacillin/tazobactam showed a broader distribution of zone diameters and MICs, with substantial overlap around the breakpoint, reflecting greater heterogeneity in susceptibility and reduced separation between susceptible and resistant isolates. Distributions for the remaining agents are shown in Figures S2–S5 (available as Supplementary data at *JAC-AMR* Online).

**Figure 1.**
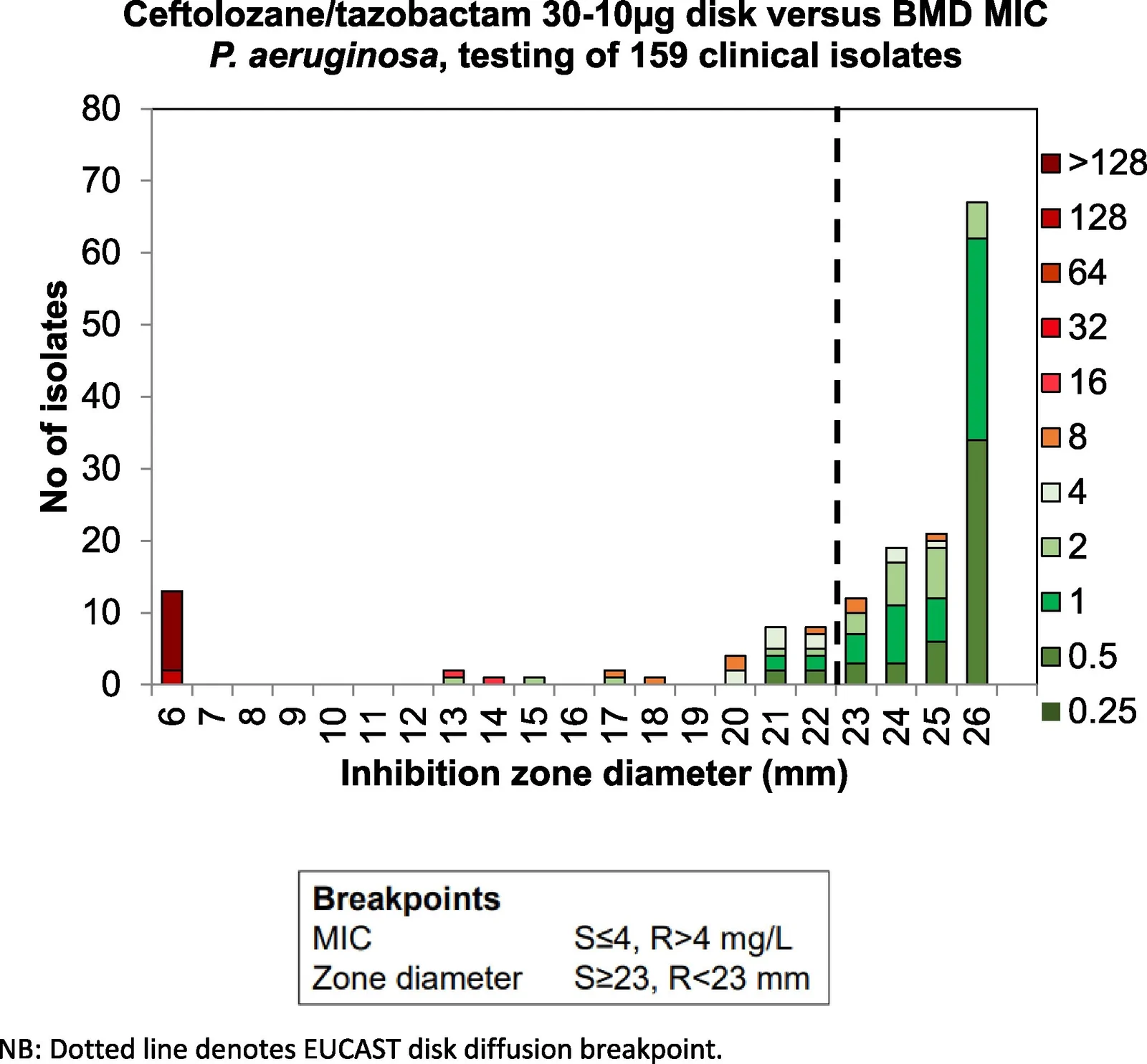
Ceftolozane/tazobactam disc zone diameter distribution versus BMD MIC. Dotted line denotes EUCAST disc diffusion breakpoint.

**Figure 2.**
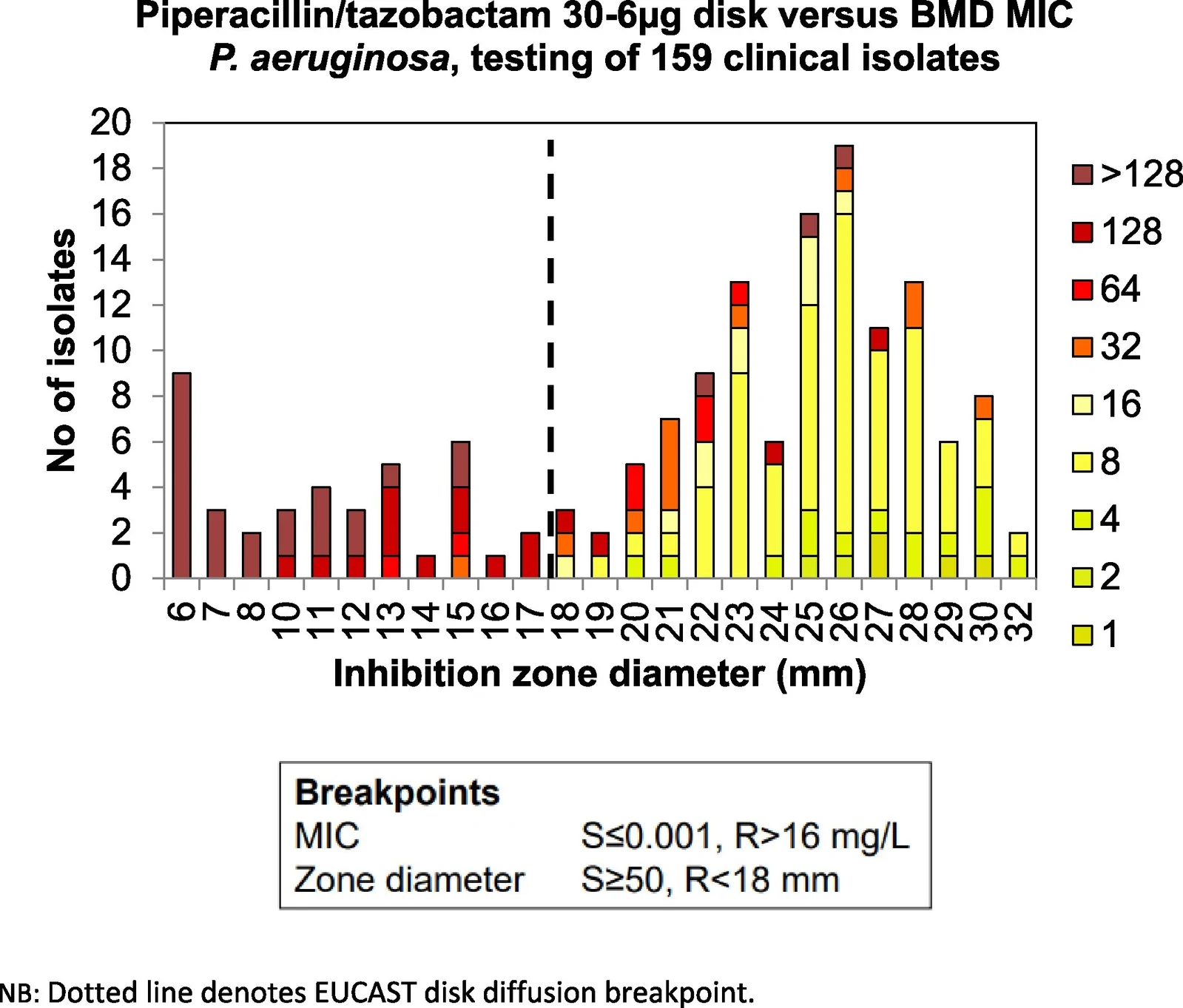
Piperacillin/tazobactam disc zone diameter distribution versus BMD MIC. Dotted line denotes EUCAST disc diffusion breakpoint.

### Discrepancies

Overall there were 25 (15.7%) discrepancies between the BMD and DD results for ceftolozane/tazobactam. Three (1.9%) VMEs (BMD = R; DD = S), resulting in false susceptibility when using DD, and 22 (13.8%) MEs (BMD = S, DD = R), resulting in false resistance when using DD, were found (Table 3). Of the 22 MEs, 7 had MICs near the breakpoint (4 mg/L or 8 mg/L); of the remaining 15, 13 had susceptible results upon retest of the DD in the reference laboratory (Tables 4 and 5).

**Table 3.** Discrepancies per agent: very major errors (VMEs) and major errors (MEs)

	C/T	TZP	CAZ	IPM	MEM	ATM
VMEs	3	19	3	7	2	6
MEs	22	1	20	9	12	10
Total errors	25	20	23	16	14	16
Total errors as % of total results (*N* = 159)	15.7	12.6	14.5	10.1	8.8	10.1

ATM, aztreonam; CAZ, ceftazidime; C/T, ceftolozane/tazobactam; IPM, imipenem; MEM, meropenem; TZP, piperacillin/tazobactam.

**Table 4.** Adjusted discrepancies with results near the breakpoint removed for ceftolozane/tazobactam and piperacillin/tazobactam

	Original VME, *n*	Retest VME, *n*	Original ME, *n*	Retest ME, *n*	% resolved upon retest
TZP	10	3	—	—	70.0
C/T	—	—	15	1	93.3

C/T, ceftolozane/tazobactam; ME, major error; TZP, piperacillin/tazobactam; VME, very major error.

**Table 5.** Retest results upon reference laboratory testing for ceftolozane/tazobactam and piperacillin/tazobactam using adjusted discrepancy results

	Adjusted results (*n)*	VME	ME	%VME	%ME
TZP	138	10	—	7.2	—
C/T	141	—	15	—	10.6

C/T, ceftolozane/tazobactam; ME, major error; TZP, piperacillin/tazobactam; VME, very major error.

For piperacillin/tazobactam, 20 (12.6%) discrepancies were observed. Nineteen (11.9%) VMEs were reported and of those, 9 had MICs near the breakpoint (16 mg/L or 32 mg/L). Of the remaining 10, 7 had resistant results upon DD retest and 3 had SIE results upon retest in the reference laboratory (Tables 4 and 5). Table S1 shows total discrepancies per lab for all six agents.

VME and ME rates were re-analysed after removing results near the breakpoints (4 mg/L and 8 mg/L for ceftolozane/tazobactam, and 16 mg/L and 32 mg/L for piperacillin/tazobactam) since erroneous interpretation would be expected to a greater degree at and around the breakpoints (Tables 4–5).

Laboratory 7 had the highest percentage (30%) of discrepant results, whereas laboratory 4 had the lowest (2.4%). It is noted that there are widespread variations in the number of isolates tested in each laboratory, which limits the ability to draw conclusions on individual laboratory performance (Table 6 and Table S1).

**Table 6.** Discrepancies according to laboratory: very major errors (VMEs) and major errors (MEs)

Laboratory	Number of isolates tested (*N* = 159)	Number of isolates tested over six agents	Number of discrepancies (VMEs and MEs)	Discrepancies (VMEs and MEs), %
1	17	108	7	6.5
2	13	78	4	5.1
3	23	138	27	19.6
4	7	42	1	2.4
5	10	60	9	15.0
6	2	12	2	16.7
7	5	30	9	30.0
8	7	42	3	7.1
9	24	144	18	12.5
10	11	66	9	13.6
11	9	54	5	9.3
12	18	108	14	13.0
13	4	24	1	4.2
14	9	54	3	5.6

## Discussion

Of the selected isolates, 86% were susceptible to ceftolozane/tazobactam by BMD. However, our findings demonstrate substantial discrepancies observed between BMD and DD results, highlighting challenges in susceptibility testing for this agent.

Discrepancies were seen in every testing laboratory and are corroborated with widespread dispersal of false susceptibility and false resistance, resulting in VMEs and MEs as observed on zone diameter versus BMD distribution histograms. A striking number of VMEs were found with piperacillin/tazobactam testing (*n* = 19), and a similarly striking number of MEs with ceftolozane/tazobactam (*n* = 22) and ceftazidime testing (*n* = 20). Combined VME and ME rates for each agent were reported as 12.6%, 15.7% and 14.5%, respectively.

If these data were part of a disc test study, these rates of VMEs and MEs would be unacceptable and therefore these data highlight widespread test performance issues of diagnostic laboratories.

Our findings particularly highlight the significant testing errors seen with using β-lactam/β-lactamase-inhibitor discs as well as ceftazidime. When discrepant isolates were retested at the reference laboratory for ceftolozane/tazobactam and piperacillin/tazobactam, VME and ME rates significantly reduced (piperacillin/tazobactam VMEs by 70.0% and ceftolozane/tazobactam MEs by 93.3%).

Adjusted VME and ME analysis for ceftolozane/tazobactam and piperacillin/tazobactam was performed to remove results from isolates near the breakpoint, which may cause a skewed analysis. However, error rates were still remarkably high at 10.6% and 7.2%, respectively. It is acknowledged that definitions of VME, ME and categorical agreement are no longer defined in ISO-20776-2 standard methodology.^8^ However, to allow comparison of MIC versus zone diameter in this dataset the use of these definitions is still needed. Therefore, acceptable rates are referenced from the CLSI AST subcommittee,^8^ which states that acceptable VME and ME rates are typically <3% of the isolates tested, with VME not surpassing <1.5%.

Acceptable ME and VME rates are typically <3% of the susceptible and resistant isolates tested, respectively. Because VMEs are of the greatest concern (i.e. false susceptibility), the FDA requires <1.5% of resistant isolates evaluated to yield VMEs.

The widespread inconsistencies found here highlight a significant issue with AST testing of piperacillin/tazobactam and ceftolozane/tazobactam specifically. Laboratories may wish to review their DD testing methods for these agents.

### Limitations

The purpose of this study was to analyse laboratory performance in interpreting AST results for various agents used in antipseudomonal therapy, chiefly ceftolozane/tazobactam and piperacillin/tazobactam. We acknowledge several limitations. The isolates chosen were biased; some isolates were purposely chosen to be at or near the breakpoint to test performance at a parameter of known interpretative difficulty. Consequently, the isolate set may not be fully representative of an unselected clinical population and the error rates observed may not directly reflect those encountered in routine diagnostic practice. However, when the results for these isolates that were close to the breakpoint were removed from VME and ME analysis, performance was still remarkably poor. Secondly, each participating laboratory used their own Mueller–Hinton agar and antibiotic discs to perform disc susceptibility, and it is expected that variances would be seen between different manufacturers. However, this variance reflects the ‘real-life’ modality in which AST is performed in NHS diagnostic microbiology laboratories and is unlikely to account for the magnitude of discrepancies seen in this report. Analysis or comparison of media and/or disc manufacturers did not form part of this study.

### Conclusions

Intrinsic and acquired resistance of *P. aeruginosa* to many antimicrobial agents remains a significant clinical concern. The ubiquity of this organism enables establishment of niches in ICUs and in immunocompromised patient cohorts, especially MDR phenotypes. Piperacillin/tazobactam is often clinically employed as first-line empirical therapy for pseudomonal infections in secondary care, with ceftolozane/tazobactam offering an important alternative for serious or MDR infection. Our ‘real-life’ multicentre performance data collected here suggest poor ability to reliably evaluate susceptibility of these agents amongst others across laboratories in England, Scotland and Wales. Clinical progress observed in patients on these agents remains a key indicator for continuing therapy, regardless of subsequent reported *in vitro* resistance. Nonetheless, the poor performance in testing disc susceptibility for these two β-lactam/β-lactamase inhibitors presents a widespread and concerning issue. Diagnostic laboratories and clinicians should be aware of the technical limitations and difficulties in performing and interpreting AST for these agents and may wish to put in place mitigating measures to improve accuracy and reliability of results.

Whilst carbapenems have traditionally been the mainstay for treating invasive infections due to *P. aeruginosa*, a drive to spare carbapenems in light of increasing resistance opens alternative treatment options with the β-lactam/β-lactamase inhibitors tested in this study. However, improvements to disc performance and interpretation are required to ensure accuracy and reliability of results for effective clinical use.

## Supplementary Material

dlag212_Supplementary_Data

## Transparency declarations

A.A.-B. was employed by MSD (UK) Limited, London, UK during the period of laboratory and data analysis and manuscript preparation (until 30 June 2024), and had shareholdings in Merck & Co., Inc., Rahway, NJ, USA during her employment at MSD (UK) Ltd, London UK. M.A. is a Trustee of the BSAC Council and is employed by MSD (UK) Limited, London, UK; he has shareholdings in Merck & Co., Inc., Rahway, NJ, USA. R.H.-G. is employed by MSD (UK) Limited, London, UK. E.C.W. is employed by MSD (UK) Limited, London, UK and has shareholdings in Merck & Co., Inc., Rahway, NJ, USA. K.B. and M.W. have nothing to declare

## Supplementary data

Figures S2 to S5 and Table S1 are available as Supplementary data at *JAC-AMR* Online.
