# Supplementary material for: INVICTUS II: comparison of disc diffusion and broth microdilution methods for susceptibility testing of ceftolozane/tazobactam and five comparator agents against a subset of INVICTUS I Pseudomonas aeruginosa clinical isolates
Source: JAC Antimicrob Resist. 2026 Oct 8;8(5):dlag212. doi: 10.1093/jacamr/dlag212 (PMC13648871; doi:10.1093/jacamr/dlag212)
Supplement: Supplementary file 1 [file dlag212_supplementary_data.docx]

S1: Total discrepancies per lab for all 6 agents; Very Major Error (VME), Major Error (ME).

|  | **C/T** | | **P/T** | | **CAZ** | | **IMI** | | **MER** | | **AZT** | | **Total discrepa-ncies per lab** |
| --- | --- | --- | --- | --- | --- | --- | --- | --- | --- | --- | --- | --- | --- |
| **Laboratory** | **VME** | **ME** | **VME** | **ME** | **VME** | **ME** | **VME** | **ME** | **VME** | **ME** | **VME** | **ME** |  |
| **01** | 0 | 3 | 0 | 0 | 0 | 0 | 0 | 0 | 0 | 2 | 1 | 2 | 8 |
| **02** | 0 | 0 | 2 | 0 | 0 | 0 | 1 | 0 | 0 | 0 | 1 | 0 | 4 |
| **03** | 0 | 5 | 1 | 1 | 0 | 9 | 3 | 2 | 0 | 1 | 1 | 4 | 27 |
| **04** | 0 | 0 | 0 | 0 | 0 | 0 | 0 | 0 | 0 | 0 | 1 | 0 | 1 |
| **05** | 0 | 1 | 4 | 0 | 0 | 2 | 0 | 0 | 0 | 1 | 1 | 0 | 9 |
| **06** | 0 | 0 | 0 | 0 | 1 | 0 | 0 | 0 | 0 | 0 | 1 | 0 | 2 |
| **07** | 0 | 0 | 3 | 0 | 0 | 0 | 2 | 1 | 1 | 2 | 0 | 0 | 9 |
| **08** | 2 | 0 | 0 | 0 | 0 | 1 | 0 | 0 | 0 | 0 | 0 | 0 | 3 |
| **09** | 0 | 3 | 3 | 0 | 2 | 2 | 1 | 2 | 0 | 4 | 0 | 2 | 19 |
| **10** | 0 | 2 | 2 | 0 | 0 | 2 | 0 | 1 | 0 | 1 | 0 | 1 | 9 |
| **11** | 1 | 3 | 0 | 0 | 0 | 0 | 0 | 0 | 1 | 0 | 0 | 0 | 5 |
| **12** | 0 | 5 | 2 | 0 | 0 | 3 | 0 | 2 | 0 | 1 | 0 | 1 | 14 |
| **13** | 0 | 0 | 1 | 0 | 0 | 0 | 0 | 0 | 0 | 0 | 0 | 0 | 1 |
| **14** | 0 | 0 | 1 | 0 | 0 | 1 | 0 | 1 | 0 | 0 | 0 | 0 | 3 |
| **Total** | 3 | 22 | 19 | 1 | 3 | 20 | 7 | 9 | 2 | 12 | 6 | 10 | 114 |
| **Combined total errors per agent** | 25 | | 20 | | 23 | | 16 | | 14 | | 16 | |  |

C/T – Ceftolozane/tazobactam, P/T – Piperacillin/tazobactam, CAZ – Ceftazidime, IMI – Imipenem, MER – Meropenem, AZT – Aztreonam

**Alt-text:** Table S1 shows the total discrepancies per lab for all 6 agents in terms of VME and ME. The highest number of combined total errors was seen for ceftolozane-tazobactam and the lowest for meropenem.

S2: Ceftazidime disk zone diameter distribution versus BMD MIC.

Ceftazidime 10µg disk versus BMD MIC P. aeruginosa, testing of 159 clinical isolates

No of isolates

| Inhibition zone diameter (mm) | 1 | 2 | 4 | 8 | 16 | 32 | 64 | 128 | >128 |
| --- | --- | --- | --- | --- | --- | --- | --- | --- | --- |
| 6 |  | 1 |  |  | 2 | 6 | 16 | 8 | 10 |
| 7 |  |  | 1 |  |  |  |  |  |  |
| 8 |  |  |  |  | 1 |  | 1 |  |  |
| 8.66667 |  |  |  |  | 1 |  |  | 1 |  |
| 9.66667 |  |  | 1 | 1 |  |  | 1 |  |  |
| 10.6667 |  | 1 |  |  | 1 |  |  |  |  |
| 12 |  | 1 |  |  | 2 |  |  |  |  |
| 13 |  |  |  | 1 |  |  |  |  |  |
| 14 |  | 1 |  | 2 |  |  |  |  |  |
| 15 |  | 3 | 1 | 1 | 1 |  |  |  |  |
| 16 |  | 1 | 2 | 2 |  |  |  |  |  |
| 17 |  |  | 1 |  |  |  |  |  |  |
| 18 |  | 1 | 2 | 1 |  |  |  | 1 |  |
| 19 |  | 4 | 3 | 1 |  |  |  |  |  |
| 20.3333 |  | 5 | 5 | 3 |  |  |  |  |  |
| 20.6667 |  | 6 | 10 |  |  | 2 |  |  |  |
| 22 |  | 10 | 6 | 2 |  |  |  |  |  |
| 23 | 1 | 8 | 3 |  |  |  |  |  |  |
| 23.6667 |  | 1 | 2 | 1 |  |  |  |  |  |
| 25.3333 |  | 5 |  |  |  |  |  |  |  |
| 26.3333 | 1 | 1 |  |  |  |  |  |  |  |
| 27 | 2 |  |  |  |  |  |  |  |  |

NB: Dotted line denotes EUCAST disk diffusion breakpoint. S ≥50, R < 17.

**Alt-text**: Figure S2 displays the distribution of ceftazidime disk diffusion (10 µg) zone diameters versus BMD MICs for 159 isolates. Resistant isolates cluster at 6 mm with high MICs while susceptible isolates cluster at 19–24 mm with low MICs. The majority of isolates fall either side of the breakpoint, with relatively few isolates occurring near the breakpoint.

S3: Imipenem disk zone diameter distribution versus BMD MIC.

Imipenem 10µg versus BMD MIC P. aeruginosa, testing of 159 clinical isolates

No of isolates

|  | 0.25 | 0.5 | 1 | 2 | 4 | 8 | 16 | 32 | 64 | 128 | >128 |
| --- | --- | --- | --- | --- | --- | --- | --- | --- | --- | --- | --- |
| 6 |  |  |  |  |  |  |  | 3 | 2 | 3 | 3 |
| 7 |  |  |  |  |  |  |  |  | 1 |  |  |
| 8 |  |  |  |  |  |  |  | 2 |  |  |  |
| 9 |  |  |  |  |  |  |  | 2 |  |  |  |
| 9.66667 |  |  |  | 1 |  |  | 3 |  |  |  |  |
| 10.6667 |  |  |  |  |  |  | 1 | 1 | 1 |  |  |
| 12 |  |  |  |  |  |  | 1 | 2 |  |  |  |
| 13 |  |  |  |  |  | 1 | 1 | 2 |  |  |  |
| 14 |  |  |  |  |  |  |  |  | 2 |  |  |
| 15 |  |  |  |  |  | 2 | 1 |  |  |  |  |
| 16 |  |  |  | 1 | 1 | 1 |  |  |  |  |  |
| 17 |  |  |  |  |  | 1 |  | 1 |  |  |  |
| 18 |  |  |  | 3 | 2 |  |  |  |  |  |  |
| 19 |  |  |  |  | 1 | 1 | 1 |  |  |  |  |
| 20.3333 |  |  |  |  | 2 | 1 |  |  |  |  |  |
| 20.6667 |  |  |  | 1 | 1 | 3 |  |  |  |  |  |
| 22 |  |  | 2 | 1 | 1 | 1 |  |  |  |  |  |
| 23 |  |  | 3 | 5 | 2 | 1 | 1 |  |  |  |  |
| 23.6667 |  |  |  | 4 | 2 |  |  |  |  |  |  |
| 25.3333 |  |  | 5 | 5 | 4 |  |  |  |  |  |  |
| 26.3333 |  | 1 | 10 | 2 | 1 |  |  |  |  |  |  |
| 27 |  | 1 | 5 | 9 | 1 |  |  |  |  |  |  |
| 28 | 1 | 1 | 5 | 6 |  |  |  |  |  |  |  |
| 29 |  | 1 | 3 | 1 | 1 |  |  |  |  |  |  |
| 30 |  | 1 | 3 | 2 |  |  |  |  |  |  |  |
| 31 |  |  | 2 | 2 | 1 |  |  |  |  |  |  |
| 32 |  | 1 | 2 |  |  |  |  |  |  |  |  |
| 34 |  |  |  |  | 1 |  |  |  |  |  |  |
| 36 |  |  | 1 |  |  |  |  |  |  |  |  |
| 40 |  |  | 1 |  |  |  |  |  |  |  |  |

NB: Dotted line denotes EUCAST disk diffusion breakpoint. S ≥20, R < 14.

**Alt-text:** Figure S3 shows the distribution of imipenem 10µg disk diffusion zone diameters versus BMD MICs for 159 isolates. Small inhibition zones (6–13 mm) are associated with high MICs, while larger zones (22–31 mm) are associated with low MICs. The majority of isolates fall either side of the breakpoint, with relatively few isolates occurring near the breakpoint.

S4: Meropenem disk zone diameter distribution versus BMD MIC

Meropenem 10µg disk versus BMD MIC P. aeruginosa, testing of 159 clinical isolates

No of isolates

| Inhibition zone diameter (mm) | 0.03 | 0.06 | 0.125 | 0.25 | 0.5 | 1 | 2 | 4 | 8 | 16 | 32 | 64 | 128 | >128 |
| --- | --- | --- | --- | --- | --- | --- | --- | --- | --- | --- | --- | --- | --- | --- |
| 6 |  |  |  |  |  |  |  |  | 1 | 3 | 8 | 7 | 2 | 2 |
| 7 |  |  |  |  | 1 |  |  |  |  |  | 1 |  |  |  |
| 8 |  |  |  |  |  |  |  |  | 2 | 1 |  |  |  |  |
| 9 |  |  |  |  |  |  |  |  |  | 1 |  |  |  |  |
| 10 |  |  |  |  |  |  |  |  | 1 | 1 | 1 |  |  |  |
| 13 |  |  |  |  |  |  | 1 | 1 | 1 | 1 |  |  |  |  |
| 14 |  |  |  |  |  |  | 1 | 1 |  |  |  |  |  |  |
| 16 |  |  |  |  |  |  |  | 4 | 1 |  |  |  |  |  |
| 18 |  |  |  |  |  |  | 1 |  |  |  |  |  |  |  |
| 19 |  |  |  |  |  |  | 2 |  |  |  |  |  |  |  |
| 20 |  |  |  |  |  | 1 |  |  | 1 |  |  |  |  |  |
| 21 |  |  |  |  |  | 2 | 2 |  |  |  |  |  |  |  |
| 22 |  |  |  |  |  | 2 | 2 |  |  |  |  |  |  |  |
| 23 |  |  |  | 1 | 2 |  |  | 1 |  |  |  |  |  |  |
| 24 |  |  |  | 2 |  | 2 | 2 |  |  |  |  |  |  |  |
| 25 |  |  |  |  | 2 | 5 |  |  |  |  |  |  |  |  |
| 26 |  |  |  | 3 | 1 | 2 | 3 |  |  |  |  |  |  |  |
| 27 |  |  |  |  | 2 | 1 |  |  |  |  |  |  |  |  |
| 28 |  |  | 1 | 5 | 5 | 1 | 2 |  |  |  |  |  |  |  |
| 29 |  |  | 1 | 2 | 2 |  | 1 |  |  |  |  |  |  |  |
| 30 |  | 1 |  | 5 | 6 |  | 1 |  |  |  |  |  |  |  |
| 31 |  |  | 1 | 3 | 2 | 2 | 1 |  |  |  |  |  |  |  |
| 32 | 1 |  |  | 6 | 4 |  | 1 |  |  |  |  |  |  |  |
| 33 | 1 | 1 | 2 | 1 | 3 | 1 |  |  |  |  |  |  |  |  |
| 34 |  |  |  | 3 |  |  |  |  |  |  |  |  |  |  |
| 35 |  |  | 1 | 3 |  |  |  |  |  |  |  |  |  |  |
| 36 |  | 1 | 1 | 1 |  |  |  |  |  |  |  |  |  |  |
| 38 |  |  |  | 1 |  |  |  |  |  |  |  |  |  |  |
| 36 |  |  | 1 |  |  |  |  |  |  |  |  |  |  |  |
| 40 |  |  | 1 |  |  |  |  |  |  |  |  |  |  |  |

NB: Dotted line denotes EUCAST disk diffusion breakpoint. S ≥20, R < 18.

**Alt text:** Figure S4 shows distribution of meropenem (10 µg disk) zone diameters versus BMD MICs for 159 isolates. Most isolates form a susceptible cluster with large inhibition zones (approximately 24–34 mm) and low MICs, while resistant isolates cluster at very small zone diameters (6–17 mm) with elevated MICs. There are few isolates near the breakpoint.

S5: Aztreonam disk zone diameter distribution versus BMD MIC.

Aztreonam 30µg disk versus BMD MIC P.aeruginosa, 159 isolates

No of isolates

| Inhibition zone diameter (mm) | 0.5 | 1 | 2 | 4 | 8 | 16 | 32 | 64 | 128 | >128 |
| --- | --- | --- | --- | --- | --- | --- | --- | --- | --- | --- |
| 6 |  |  |  |  | 1 |  |  | 3 | 14 | 1 |
| 8 |  |  |  |  |  |  |  | 2 | 1 |  |
| 9 |  |  |  |  |  | 1 |  | 1 |  |  |
| 10 |  |  |  |  |  |  |  | 1 |  |  |
| 11 |  |  |  |  |  |  |  | 2 |  |  |
| 12 |  |  |  |  |  |  | 2 | 2 | 2 |  |
| 13 |  |  |  |  |  |  | 3 |  |  |  |
| 14 |  |  |  |  |  | 1 | 4 | 1 |  |  |
| 15 |  |  |  |  | 1 | 2 | 3 |  | 1 | 2 |
| 16 |  |  |  |  | 2 | 1 | 1 | 1 | 2 |  |
| 17 |  |  |  |  |  | 1 | 1 |  |  | 2 |
| 18 |  |  |  |  |  |  | 2 |  |  |  |
| 19 |  | 1 |  |  | 1 |  |  |  |  |  |
| 20 |  |  |  | 1 | 4 | 2 |  |  | 1 |  |
| 21 |  |  |  |  | 10 |  |  |  |  |  |
| 22 |  |  |  | 1 | 11 | 1 | 1 |  |  |  |
| 23 | 1 |  |  |  | 15 | 5 |  |  |  | 1 |
| 24 |  | 1 |  | 2 | 7 |  |  |  |  |  |
| 25 |  |  |  | 2 | 8 |  |  |  |  |  |
| 26 |  |  |  |  | 3 |  |  |  |  |  |
| 27 | 1 |  |  | 1 |  |  |  | 1 |  |  |
| 28 |  | 1 |  | 3 | 2 | 1 |  |  |  |  |
| 29 |  |  |  |  | 1 |  |  |  |  |  |
| 30 |  |  |  | 1 |  | 1 |  |  |  |  |
| 34 | 1 |  | 2 |  |  |  |  |  |  |  |

NB: Dotted line denotes EUCAST disk diffusion breakpoint. S ≥50, R < 18.

**Alt text:** Figure S5 shows distribution of aztreonam (30 µg disk) zone diameters versus BMD MICs for 159 isolates. Isolates are distributed across a wide range of zone diameters, with a susceptible cluster centred around 20–25 mm and a resistant cluster at smaller diameters (6–17 mm) with higher MICs. Several isolates occur near the breakpoint.
